# The Effect of Weight-Loss Interventions on Cervical and Chin Subcutaneous Fat Depots; the CENTRAL Randomized Controlled Trial

**DOI:** 10.3390/nu13113827

**Published:** 2021-10-27

**Authors:** Gal Tsaban, Avital Bilitzky-Kopit, Anat Yaskolka Meir, Hila Zelicha, Yftach Gepner, Ilan Shelef, Omri Orr, Yoash Chassidim, Benjamin Sarusi, Uta Ceglarek, Michael Stumvoll, Matthias Blüher, Meir J. Stampfer, Iris Shai, Dan Schwarzfuchs

**Affiliations:** 1 Faculty of Health Sciences, Ben-Gurion University of the Negev, Beer-Sheva 84105, Israel; avitalbilitzky@gmail.com (A.B.-K.); anatyas@gmail.com (A.Y.M.); hila.zelicha@gmail.com (H.Z.); omriorr@gmail.com (O.O.); irish@bgu.ac.il (I.S.); 2 Department of Cardiology, Soroka University Medical Center, Beer-Sheva 84101, Israel; 3 Department of Epidemiology and Preventive Medicine, Sackler Faculty of Medicine, and Sylvan Adams Sports Institute, School of Public Health, Tel-Aviv University, Tel-Aviv 96678, Israel; gepner@tauex.tau.ac.il; 4 Division of Clinical Radiology, Soroka University Medical Center, Beer-Sheva 84101, Israel; shelef@bgu.ac.il; 5 Research Center, Soroka University Medical Center, Beer-Sheva 84101, Israel; yoash.chassidim@gmail.com; 6 Department of Medicine, Nuclear Research Center Negev, Dimona 84190, Israel; benny@rotemi.co.il (B.S.); dan.nurit@gmail.com (D.S.); 7 Department of Medicine, University of Leipzig, 04103 Leipzig, Germany; Uta.Ceglarek@medizin.uni-leipzig.de (U.C.); Michael.Stumvoll@medizin.uni-leipzig.de (M.S.); Matthias.Blueher@medizin.uni-leipzig.de (M.B.); 8 Channing Division of Network Medicine, Department of Medicine, Brigham and Women’s Hospital, Harvard T.H. Chan School of Public Health, Boston, MA 02115, USA; STAMPFER@hsph.harvard.edu; 9 Emergency Medicine Division, Soroka University Medical Center, Beer-Sheva 84101, Israel

**Keywords:** cervical subcutaneous fat, chin subcutaneous fat, dietary intervention, weight loss, magnetic resonance imaging, insulin resistance

## Abstract

Accumulation of cervical and chin subcutaneous adipose tissues (SAT) represent known phenotypes of obesity. We aimed to evaluate the sensitivity of these fat storages to long-term weight-loss directed lifestyle-intervention and to assess their relations to bodily-adiposity, insulin-resistance, and cardiometabolic risk; We randomly assigned 278 participants with abdominal-obesity/dyslipidemia to low-fat or Mediterranean/low-carbohydrate diets +/− physical-activity. All participants underwent an 18 month whole-body magnetic resonance imaging follow-up, from which we assessed cervical and chin SAT-areas; Participants (age = 48 years; 90% men; body-mass-index = 30.9 kg/m^2^) had an 18-month adherence-rate of 86%. Cervical-SAT and chin-SAT decreased after 6-months (−13.1% and −5.3%, respectively, *p* < 0.001). After 18-months only cervical-SAT remained decreased compared to baseline (−5%, *p* < 0.001). Cervical and chin-SAT 18-month changes were associated with changes in weight (r = 0.70, r = 0.66 respectively; <0.001 for both) and visceral-adipose-tissue (VAT; r = 0.35, r = 0.42 respectively; <0.001 for both). After adjustment to VAT, waist-circumference, or weight-changes, chin-SAT 18-month reduction was associated with favorable changes in fasting-glucose (β = 0.10; *p* = 0.05), HbA1c (β = 0.12; *p* = 0.03), and homeostasis-model-assessment-of-insulin-resistance (β = 0.12; *p* = 0.03). Cervical-SAT 18-month reduction was associated with decreased triglycerides (β = 0.16; *p* = 0.02) and leptin (β = 0.19; *p* = 0.01) independent of VAT; Cervical and chin-SATs are dynamic fat depots that correspond with weight-loss and are associated with changes in cardiometabolic profile. In long-term, chin-SAT displays a larger rebound compared with cervical-SAT. Chin-SAT accumulation is associated with in insulin-resistance, independent of central obesity. (ClinicalTrials identifier NCT01530724)

## 1. Introduction

The cervical subcutaneous adipose tissue (cervical-SAT) is a unique portion of the upper SAT located between the skin dermis and the cervical fascia [1]. The associations of visceral-adipose-tissue (VAT) with cardiovascular disease (CVD) risk factors are well-established [2], yet the role of SAT accumulation remains unclear [3,4,5].

SAT is a highly heterogeneous fat depot. In the trunk region, deep-SAT strongly correlates with cardiometabolic risk [6], while superficial-SAT is associated with improved metabolic state [7]. While lower body SAT (gluteofemoral-fat) is considered protective, upper body SAT accumulation is more pathogenic [8]. Prior studies demonstrated associations of upper trunk SAT with insulin resistance and an adverse lipid profile [6,9,10]. Upper body SAT accounts for most circulating free fatty acid concentrations [11], supporting its role in insulin resistance and CVD risk [5,8].

Cervical-SAT accumulation, traditionally estimated by neck circumference, has been associated with CVD risk factors beyond body-mass index (BMI) and VAT [12,13,14]. An extreme example of cervical-SAT accumulation is the “Buffalo hump” phenomenon (a pathological enlargement of dorsocervical-SAT, which was suggested as a phenotypic marker for metabolic syndrome and insulin resistance [15,16]). Computed tomography (CT) or MRI enables the distinction between deep and subcutaneous cervical-SAT [17]. Thus, they aid in defining their specific association with the cardiometabolic state. Prior cross-sectional studies reported significant associations of cervical-SAT with BMI, waist circumference (WC), SAT, VAT, hepatic steatosis, insulin resistance, dyslipidemia, and metabolic syndrome [1,18,19]. Albeit, longitudinal data regarding the sensitivity of cervical subcutaneous fat to weight loss-directed lifestyle interventions and its independent association with cardiometabolic risk are limited.

Fat accumulation in the submental region, i.e., “double chin”, is typical in obese and elderly populations. Excess fat in this unique depot has been suggested to correlate with diabetes, metabolic syndrome, and obesity-sleep-apnea [15,20,21]. Nevertheless, the exact role of chin-SAT as an indicator for insulin resistance and metabolic syndrome and its flexibility regarding weight loss remains to be determined.

The 18-months CENTRAL randomized controlled trial [22] aimed to determine the impact of different weight-loss-directed lifestyle strategies on the distribution of body fat depots. Here, we report the effect of different lifestyle modification strategies on cervical and chin SAT depots dynamics. As well, we aimed to examine these storage pools association with obesity and cardiometabolic state. Our a-priori hypothesis was that cervical-SAT represents a pathogenic fat depot that can be modified by lifestyle intervention, while chin-SAT is a unique fat pool conferring high metabolic risk beyond obesity.

## 2. Materials and Methods

### 2.1. Eligibility and Study Design

The CENTRAL randomized controlled trial [22] (clinicaltrials; NCT01530724) is an 18-month intervention trial conducted between October 2012 and April 2014 in the Nuclear Research Center Negev, Dimona, an exclusive workplace with an on-site medical clinic and cafeteria. The criteria for eligibility were abdominal obesity (WC > 102 cm for men, >88 cm for women), or combination of high serum triglycerides (>150 mg/dL) and low high-density lipoprotein cholesterol (HDL-c < 40 mg/dL for men and <50 mg/dL for women). The exclusion criteria were: serum creatinine ≥2 mg/dL, disturbed liver function test (≥3 fold the upper level of the enzymes ALT and AST), pregnancy or lactation, active cancer, inability to start physical activity in the gym, or high physical activity (≥3 h/week) and participation in another trial.

**Figure 1:**
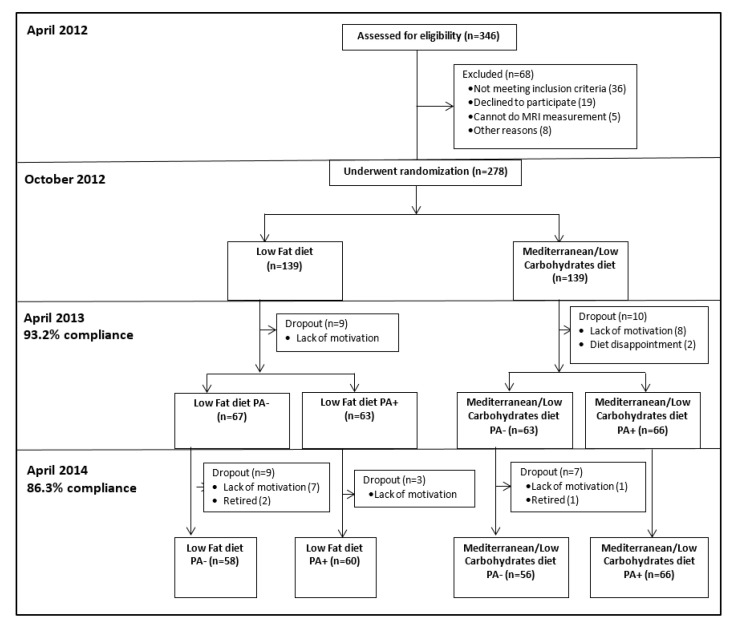
Flowchart of the CENTRAL trial.

The randomization was executed in two phases. First, the participants were randomized to one of two diet groups: Low-fat diet (LF) [23] and Mediterranean/low-carbohydrate diet (MED/LC) [24]. Second, after 6-months of dietary intervention, the two dietary groups were further randomized into diet only (LF PA−, MED/LC PA−) or diet plus physical activity (PA) interventions (LF PA+, MED/LC PA+). The randomization was performed with an allocation ratio of 1:1 to the two treatment groups, within strata of baseline VAT area for the first randomization, and the residence address (to ensure similar geographical distance from the gym) in the second randomization (in blocks of two). Participants were randomized in one phase after their strata characteristics were defined by random number sequencing. The study protocol was approved by the Medical Ethics Board and the Helsinki Committee of the Soroka University Medical Center. All the participants provided written informed consent and received no financial compensation or gifts. Details regarding the interventions are provided in Supplemental Material File S1. A flowchart of the CENTRAL trial is provided in Figure 1.

### 2.2. Outcomes

#### 2.2.1. Magnetic Resonance Imaging

We performed a whole-body 3-Tesla MRI (Ingenia 3.0 T, Philips Healthcare, Best, the Netherlands) to scan all participants at baseline and after 18 months of intervention. For technical reasons (MRI availability), at 6 months, we performed an additional scan on a random subset of 155 participants from both dietary arms. The scanner utilized a 3D modified Dixon imaging technique (2 mm thickness and 2 mm spacing), fast-low-angle shot (FLASH) sequence with a multi-echo two-excitation pulse sequence for phase-sensitive encoding of fat and water signals (TR, 3.6 ms; TE1, 1.19 ms; TE2, 2.3 ms; FOV, 520 × 440 × 80 mm; 2 × 1.4 × 1 mm voxel size). Four images of the phantoms were generated, including in-phase and out-of-phase, fat, and water phase.

**Figure 2:**
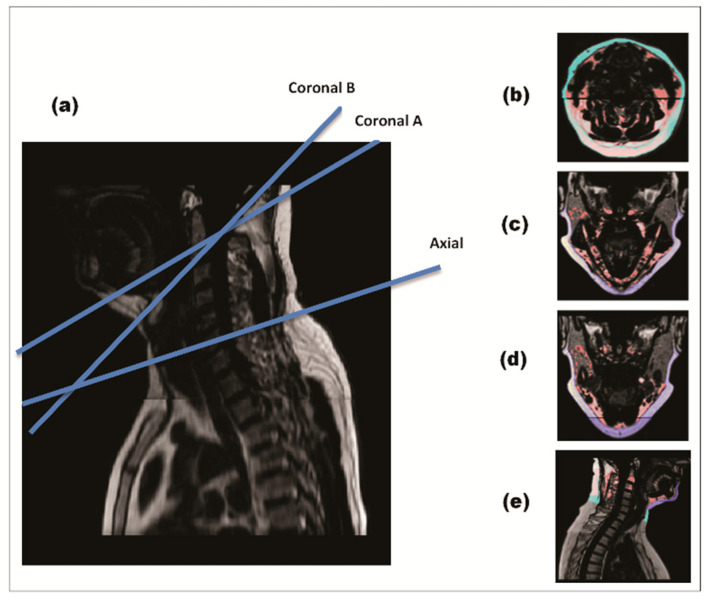
MRI of cervical-subcutaneous-adipose-tissue. Four anatomical slices per subject were extracted for each time point in each time. During quantification, cervical-SAT was divided into two sub-compartments—chin-SAT (dark blue) and cervical-SAT (light blue). Image (**a**) demonstrates a mid-sagittal section of the cervical region with blue lines placing the two coronal and axial sections. Image (**b**) demonstrates an axial slice, Image (**c**) coronal A slice, Image (**d**) coronal B slice and Image (**e**) sagittal slice.

Cervical and chin SAT were quantified using a semiautomatic MATLAB-based program. A threshold value (0.4) was defined to distinguish between adipose and lean tissues automatically. A total of four MRI slices per subject were extracted in each time period (Figure 2). The slices were chosen in three anatomical planes: axial plane, sagittal plane, and two coronal planes. We extracted the slices using specific, pre-selected anatomical landmarks: Axial slice: at the C4-C5 intervertebral disk (IVD) level. This anatomical landmark was parallel to the area of measurement tape placement in previous studies measuring neck circumference [1,12]. Coronal slices: in two planes connecting the tip of dens with: the most internal surface of the anterior mandible (coronalA); the midpoint between the internal surface of the anterior mandible and the anterior longitudinal ligament of the vertebrae (coronalB); Sagittal slice: in the mid sagittal plane, between the tip of the dens (C2) superiorly and C6-C7 IVD inferiorly.

Cervical-SAT in each slice was defined as the fat tissue that lies between the skin externally and the cervical fascia and muscles internally. The MRI scan allows visualization of the cervical fascia, and the exact delineation of cervical-SAT contour was performed manually. Chin SAT was measured in mid-sagittal and coronal sections, and cervical-SAT was measured in mid-sagittal and axial sections. Fat quantification was performed blinded to time point and group treatment. Inter-rater correlation was measured in 48 images (12 for each section) with interclass-correlation of 0.996 for the axial section, 0.967 for the sagittal section, 0.996 for the coronalA section, and 0.989 for the coronalB section. The assessment of other fat depots is detailed in Supplemental Material File S2.

#### 2.2.2. Clinical and Anthropometric Outcomes

Height was measured to the nearest millimeter using a standard wall-mounted stadiometer. Bodyweight was measured monthly without shoes to the nearest 0.1 kg. WC was measured midpoint between the last rib and the iliac crest to the nearest millimeter by a standard procedure using a 150-cm anthropometric measuring tape. Blood pressure was measured using an automated system (Datascop acutor 4), following 5 min of rest. Blood and urine samples were taken at 8 a.m. after a 12-h fast. The samples were centrifuged and stored at −80 °C with a backup system. All biochemical analyses—lipids, glycemic, inflammatory, and liver biomarkers—were performed simultaneously in the Leipzig University, Germany (laboratory methods detailed in Supplemental Material File S3).

#### 2.2.3. Statistical Analysis

The CENTRAL trial primary outcomes were (1) changes in body fat composition, (2) changes in weight, and (3) changes in waist circumference. The main results of the study were previously reported elsewhere [22]. The specific aim of the present analysis is to thoroughly describe and address the 18-month changes in cervical and chin subcutaneous fat compartments during lifestyle interventions and their associations with cardiometabolic risk at baseline and following weight loss. The primary outcome of this analysis was 18-months changes of cervical and chin SATs which were compared within and across dietary intervention groups at 6-months and dietary and PA intervention groups at 18-months. Changes in anthropometric and cardiometabolic parameters and their association with cervical and chin SATs were secondary outcomes. Power calculations are provided in Supplemental Material File S4. Cervical-SAT was represented by 3 indices: (1) Total cervical-SAT- calculated by summing the fat of axial and sagittal sections with the mean fat of the two coronal sections; (2) Chin-SAT- calculated by summing chin-SAT in the sagittal section with the mean chin-SAT of the two coronal sections; and (3) Cervical-SAT- calculated by summing cervical-SAT in the sagittal section with the cervical-SAT in the axial section. Baseline characteristics of the two diet groups were compared using independent samples T-test for quantitative variables or chi-square test for categorical data. Kendall’s tau-b correlation test was used to assess the *p*-of-trend for baseline parameters across sex-specific tertiles of BMI. The six and 18-month changes of cervical-SAT within each intervention group were assessed by paired sample T-test. An intention-to-treat (ITT) analysis was performed to assess the differences between intervention groups, accounting for all participants primarily recruited for the study and underwent MRI with missing data completion using the most recent values of cervical-SAT, according to the last-observation-carried-forward method. This was done, as described before [22,25,26], to ensure avoidance of bias due to differences in drop-out rates and participants’ adherence to their allocated intervention.Differences between the two diet groups were assessed using independent samples T-test or Mann-Whitney U test, as appropriate. Differences between the four intervention groups were assessed by one-way analysis of variance (ANOVA) or Kruskal–Wallis test. Univariate associations between 18-month changes of cervical-SAT and changes of blood biomarkers, anthropometric parameters, and other fat depots were analyzed using Pearson correlation coefficients. Multivariate linear regression models were used to identify independent factors associated with cervical-SAT dynamics. Models were adjusted for age and sex, and 18-month WC changes, 18-month VAT changes, or 18-month weight changes (each one at a time).

Data were reported as means ± standard-deviations for quantitative variables or frequencies for categorical variables. The distribution of parameters was tested for normality using the Shapiro-Wilk test. Non-normally distributed parameters were log-transformed to achieve normal distribution before statistical analyses. The data were analyzed using IBM SPSS version 26 (Armonk, NY, USA). All *p*-values were two-sided and statistical significance was set at *p* ≤ 0.05.

## 3. Results

### 3.1. Baseline Characteristics

At baseline, 273 of the 278 participants of the CENTRAL study were eligible for cervical and chin SAT quantification. Five subjects were excluded due to the suboptimal quality of the MRI scan. The mean age was 47.8 ± 9.3 years, the mean BMI was 30.9 ± 3.7 kg/m^2,^ and the mean WC was 106.7 ± 9.4 cm. Most of the participants (89.7%) were men. The mean cervical-SAT was 33.34 ± 12.2 cm^2^ for men, and 34.47 ± 15.3 cm^2^ for women (*p* = 0.653), and the mean chin-SAT was 17.74 ± 4.69 cm^2^ for men and 17.77 ± 3.34 cm^2^ for women (*p* = 0.975).

Baseline characteristics of the participants across sex-specific tertiles of BMI are presented in Table 1. Baseline cervical-SAT and chin-SAT were correlated with BMI tertiles (*p* of trend < 0.001 for all). Higher baseline cervical and chin-SAT correlated with increased WC, diastolic and systolic blood pressure, leptin, insulin, HbA1C, and homeostasis-model-assessment-of-insulin-resistance (HOMA-IR; *p* < 0.05 for all). Cervical-SAT also positively correlated with age (*p* < 0.05). Higher VAT, superficial-SAT, and deep-SAT were associated with higher cervical and chin SATs (*p* < 0.001 for all). At baseline, intervention groups had similar anthropometric characteristics, blood biomarkers, and body fat depots levels (Supplemental Material Table S1).

### 3.2. Changes in Cervical and Chin SATs during the Lifestyle Intervention

**Figure 3:**
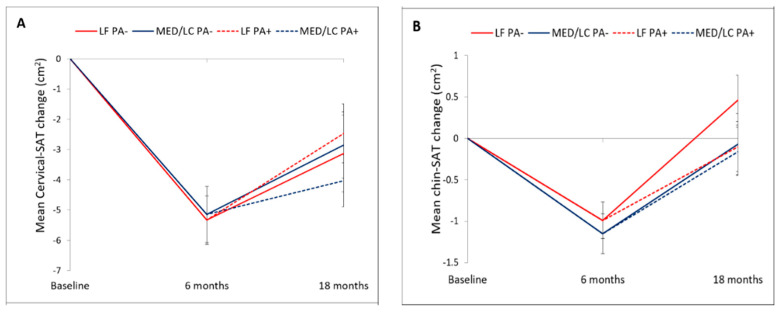
18 months cervical and chin SATs trajectories across lifestyle intervention groups. Panel (**A**) presents the dynamics of cervical-SAT and panel (**B**) dynamics of chin-SAT. Vertical bars indicate standard errors. There were no statistically significant differences between the intervention groups. LF, low-fat; MED/LC, Mediterranean/low-carbohydrate-; PA, physical activity.

Adherence to the trial was 93.2% at 6-month and 86.3% at 18-month (Figure 1). Mean weight loss was −5.4 ± 4.7 kg after 6-month, and −3.0 ± 5.5 kg after 18-month (*p* < 0.001 vs. baseline for both), with no significant differences between the intervention groups. Out of 240 participants who completed the 18-month trial, images of 217 participants were eligible for fat quantification. The 18-month changes in cervical and chin SATs across the four intervention groups are detailed in Table 2 and Figure 3. After 6-months of intervention, cervical-SAT (−13.1 ± 16.9%) and chin-SAT (−5.3 ± 10.7%) had reduced significantly (*p* < 0.001 for both) and similarly across intervention groups. Corresponding to body weight regain, a trend of regain in cervical-SAT was observed between 6 to 18-month of intervention. After 18-month of intervention, cervical-SAT decreased by −7.8 ± 18.7% (*p* < 0.001) similarly across intervention groups, while changes in chin-SAT were not significant.

### 3.3. Association between 18-Month Changes in Cervical-SAT with Changes in Anthropometric Measurements and Body Fat Depots

**Figure 4:**
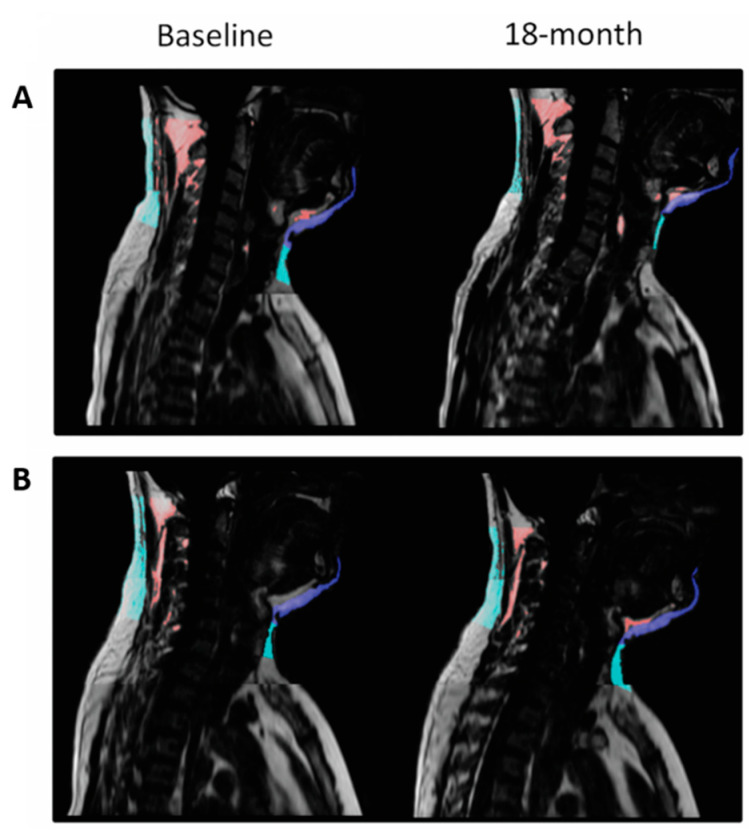
MRI of cervical-SAT—comparison between two matched participants with different weight loss achievements. Panel (**A**) and (**B**) demonstrate the sagittal section of subjects A and B, respectively. Cervical-SAT was divided into color-coded compartments: light blue, cervical-SAT; dark blue, chin-SAT; and red, non-classified fat- fat beneath the fascia. Both men had a baseline weight of 83 kg and waist circumference of 105 cm; following the 18-month intervention, participant A lost 18 kg, and participant B gained 5.4 kg. The two men differed distinctly in their 18-month cervical-SAT (−51.97% vs. +2.52%) and chin-SAT (−27.5% vs. +4.82%) reductions.

In models adjusted for age and sex, the 18-month decrease of cervical and chin SATs were positively associated with the 18-month decrease of weight (β = 0.697 and β = 0.664, respectively; *p* < 0.001 for both) and WC (β = 0.487 and β = 0.509; *p* < 0.001 for both) (Supplementary Material Table S2). We also assessed the associations between cervical-SAT and other body fat depots dynamics with the hypothesis that changes in cervical-SAT might represent an external marker for changes in visceral and ectopic fat depots. In multivariate models, cervical and chin SATs dynamics directly correlated with the dynamics of superficial-SAT, deep-SAT, and VAT (*p* < 0.001 for all). VAT changes produced the strongest association with both cervical and chin dynamics (VAT: β = 0.352 and β = 0.415, respectively; Superficial-SAT: β = 0.275 and β = 0.347; Deep-SAT: β = 0.284 and β = 0.331). In addition, both cervical and chin SATs dynamics were positively associated with the dynamics of hepatic fat, renal sinus fat, intermuscular fat, and pericardial fat (*p* < 0.05 for all) also after adjustment to VAT changes. An illustrative example of cervical and chin SATs dynamics in two participants with different weight changes is presented in Figure 4.

### 3.4. Association between 18-Month Changes in Chin-SAT with Changes in Cardiometabolic Profile

**Figure 5:**
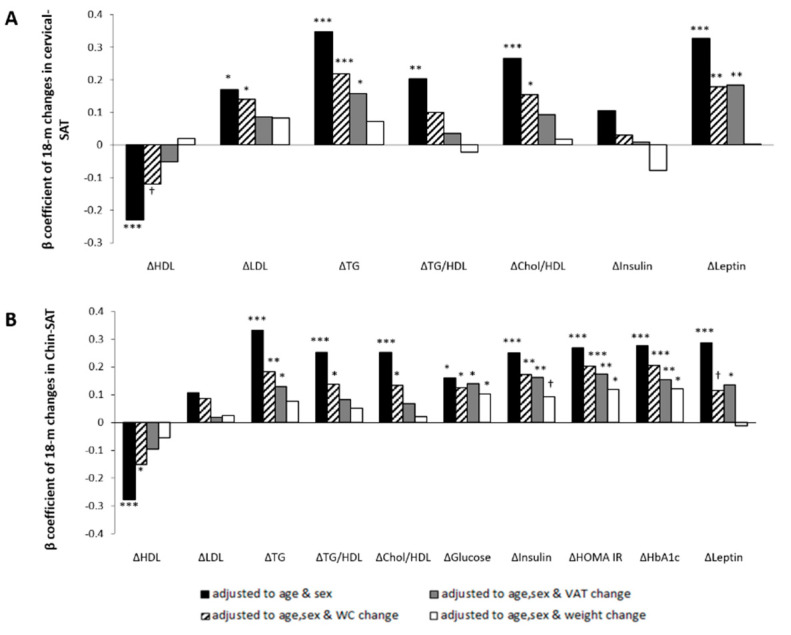
Associations between 18-month changes in cervical and chin SATs and changes of blood biomarkers; multivariate linear regression models. Panel (**A**) and panel (**B**) show cervical-SAT and chin-SAT dynamics associations, respectively. Models were adjusted for age and sex (black bar); age, sex and WC change (black with white strips bar); age, sex, and VAT change (grey bar); and age, sex, and weight change (white bar). Statistical significance: *** *p* ≤ 0.001; ** *p* ≤ 0.01; * *p* ≤ 0.05; ^†^ *p* ≤ 0.1. WC, Waist circumference; VAT, visceral adipose tissue; HDL-c, high-density lipoprotein cholesterol; LDL-c, low-density lipoprotein cholesterol; TG, triglycerides; HOMA, homeostasis model assessment; IR, insulin resistance; HbA1c, glycated hemoglobin.

Chin-SAT reduction was associated with improved glycemic and lipid biomarkers in multivariate linear regression models adjusted for age and sex (Figure 5A). To assess whether chin-SAT reduction was associated with cardiometabolic risk beyond visceral obesity, models were also adjusted for 18-month changes in WC or VAT. In multivariate models adjusted for age, sex and 18-month changes in VAT, chin-SAT reduction was associated with 18-month decrease of TG (β = 0.128; *p* = 0.05), glucose (β = 0.139; *p* = 0.017), insulin (β = 0.162; *p* = 0.007), HbA1c (β = 0.155; *p* = 0.011), HOMA-IR (β = 0.174; *p* = 0.004) and leptin (β = 0.136; *p* = 0.037). In addition, models were adjusted for 18-month changes in weight to examine whether the association between chin-SAT reduction and cardiometabolic risk was mediated by the weight reduction during the intervention. In models adjusted for age, sex and 18-month changes in weight, chin-SAT reduction remained significantly associated with 18-month decrease of glucose (β = 0.102; *p* = 0.05), HbA1c (β = 0.121; *p* = 0.028) and HOMA-IR (β = 0.118; *p* = 0.034), yet the association with insulin was (β = 0.092; *p* = 0.098).

### 3.5. Associations between 18-Month Changes in Cervical-SAT with Changes in Cardiometabolic Profile

In multivariate linear regression models adjusted for age and sex, cervical-SAT reduction was associated with improved lipid profile (Figure 5B). In models adjusted for age, sex and 18-month WC change, cervical-SAT reduction was associated with 18-month decrease of LDL-c (β = 0.140; *p* = 0.03), TG (β = 0.218; *p* = 0.001), cholesterol/HDL-c ratio (β = 0.154; *p* = 0.021), leptin (β = 0.179; *p* = 0.014) and leptin/adiponectin ratio (β = 0.178; *p* = 0.007). In models further adjusted for 18-month changes in VAT, cervical-SAT reduction was associated with 18-month decrease of TG (β = 0.158; *p* = 0.021), leptin (β = 0.185; *p* = 0.007) and leptin/adiponectin ratio (β = 0.135; *p* = 0.036). These associations were attenuated in models adjusted for 18-month weight changes.

## 4. Discussion

In this 18-month trial, we examined the effect of lifestyle interventions on changes of cervical and chin SATs using whole-body MRI. During the intervention, cervical-SAT dynamics paralleled weight changes but were not differentially altered by the intervention groups. Chin-SAT reduced after the 6-month rapid weight loss phase but was regained in parallel to the weight regain phase. While chin-SAT reduction was associated with improved glycemic biomarkers beyond weight changes, the associations of cervical-SAT reduction with cardiometabolic biomarkers were mediated by weight loss. Although located superficially, both cervical and chin SATS were closely related to abdominal visceral fat loss and reduction of ectopic fat in several storage pools. These findings offer insight into potential metabolic roles of specific SAT accumulation and the significance of cervical and chin SATs as surface markers for metabolic changes during weight loss intervention.

The present study has several limitations. First, the small number of women, a derivative of the gender profile in the unique workplace in which the study was performed, limits our ability to apply results to both genders. Second, the measurements of cervical-SAT were semiautomatic and therefore prone to user-dependent errors. However, we achieved high inter and intra-rater correlations (>0.97), ensuring our measurements’ reliability. There is no gold standard measurement of cervical-SAT, which limits our ability to assess the measurement of validity. We set the guidelines of fat extraction and quantification according to clear anatomical landmarks to enable maximal reproducibility and accurate measurements. Finally, total lean body mass or fat mass measurements were not available from our MRI analysis. The strengths of the study include the use of 3-Tesla MRI, which is considered the gold standard technique to quantify fat, the relatively long follow-up duration and large sample size of the study, and the high rate of adherence.

Cervical-SAT accumulation has been previously associated with insulin resistance [12,13,14,19], metabolic syndrome [1,12], and other cardiovascular risk factors [18,27]. A recent study examined cervical-SAT accumulation in 3 compartments (subcutaneous, posterior, and perivertebral) and reported associations of cervical-SAT with metabolic syndrome in both men and women, and fasting glucose, HDL-c, and TG in women only [1]. Accordingly, it was suggested that the subcutaneous compartment is associated with an adverse metabolic profile. Our study focused solely on the subcutaneous fat and examined two regions of fat accumulation- cervical and chin areas. We observed different cervical and chin SAT associations with metabolic profile, with chin-SAT being more consistently associated with cardiometabolic risk. According to our findings, the cervical-SAT reduction was associated with decreases of TG, leptin, and leptin/adiponectin ratio, even after adjustment for VAT. However, these associations were attenuated after accounting for weight loss. The lack of independent associations with lipid and glycemic profiles might reflect the small number of women in our study, as prior studies suggested cervical-SAT accumulation being more sensitive to cardiometabolic risk in women [1,12,14]. In addition, the differences in the methods of cervical-SAT measurements, including imaging technique, the definition of the cervical area, and guidelines for fat quantification [1,18,19], might explain this inconsistency.

Our results suggest that chin-SAT changes might be an indicator of glycemic profile. These findings are consistent with prior studies demonstrating associations of upper body SAT with insulin resistance [6,9,19]. In addition, cross-sectional studies of Asian Indian populations have suggested that the presence of “double chin” may signify a heightened risk for metabolic syndrome16 and non-alcoholic fatty liver disease [28]. Another study among patients with familial partial lipodystrophy have suggested an association between chin-SAT and the risk of developing diabetes, as women with diabetes had significantly increased skinfold thickness at the chin compared with women without diabetes. However, this association was not adjusted for BMI [20]. These studies, along with ours suggest the role of chin-SAT as a phenotypic marker for insulin resistance.

We propose several mechanisms to explain the observed associations between chin-SAT changes and insulin resistance. First, hypertrophy of adipose tissue in the facial and cervical area, including “double chin”, “moon face” and “buffalo hump”, as observed in Cushing’s syndrome, might be a consequence of excessive exposure to glucocorticoids [29]. A recent study suggested that changes in cortisol metabolism may explain the association between cervical-SAT and glycemic profile [19,30]. Second, chin-SAT may be a proxy for VAT in the cervical region. A close relationship between the chin and visceral adiposity is suggested by observations of lipodystrophy syndrome in HIV-infected patients, characterized by dorsocervical and submandibular fat accumulation and visceral obesity [31]. The concomitant development of chin and visceral fat in these patients suggests that these two distinct fat depots might accumulate in tandem and have similar metabolic properties. Third, according to the hypothesis proposed by Sierra-Johnson et al., facial fat is an active source of free fatty acids that directly, or through its relationship with VAT, may produce impaired glucose tolerance [32].

The precise biological activity of cervical-SAT is not fully understood. It has been shown that upper body subcutaneous fat is responsible for most of the systemic free fatty acids release [11]. Free fatty acids excess has been associated with insulin resistance and increased hepatic very-low-density lipoprotein (VLDL) production [11,12]. In different context, cervical-SAT as measured by neck circumference was associated with increased secretion of pro-inflammatory cytokines which in turn increases insulin resistance [33]. Moreover, cervical-SAT accumulation may provoke obstructive sleep apnea syndrome, which plays an important role in the development of CVD [17,34]. In contrast, recent studies reported the presence of brown adipose tissue (BAT) in human’s cervical and supraclavicular area [35]. BAT is associated with healthy phenotype via its unique thermogenic activity and its capacity for combusting lipids and carbohydrate [36]. However, active BAT is lower in patients with obesity or type 2 diabetes [37]. In addition, the cervical-SAT depots are not homogenous, the deep cervical-SAT has characteristics of BAT while the subcutaneous fat is a white depot [35]. The presence of BAT/beige fat in cervical regions might explain the conflicting results regarding cervical-SAT and cardiometabolic risk. Our current report sheds light on the possible independent role of cervical and chin SATs in promoting insulin resistance and cardiometabolic imbalances associated with obesity. The results of the study suggest that regression of these unique SATs is not only weight loss sensitive but also reflects the recovery of sensitivity to insulin beyond the effect of weight or visceral adipose tissue and therefore contributes to the understanding of the importance of addressing the existence of excessive cervical and chin SATs in obesity metabolic phenotyping.

## 5. Conclusions

In conclusion, we followed the dynamics of two specific SAT depots in the cervical and chin regions during long-term weight loss directed lifestyle intervention. Changes in these two SAT depots corresponded with weight trajectory. While, in long-term, cervical-SAT remained significantly reduced, chin-SAT was more prone for a large rebound. Chin-SAT reduction was independently associated with an improved glycemic profile, possibly suggesting a novel role of chin-SAT in obesity-related insulin resistance.

## Figures and Tables

**Table 1:** Baseline characteristics of the study population across sex-specific tertiles of BMI.

	Low Tertile (*n* = 91)	Intermediate Tertile (*n* = 90)	Top Tertile (*n* = 92)	*p*-of-Trend	Entire (*n* = 273)
BMI (Kg/m^2^)					
Men (*n* = 245)	24.04–29.23	29.24–31.93	31.94–43.47		
Women (*n* = 28)	24.06–27.02	27.03–31.72	31.73–42.28		
Chin-SAT (cm^2^)	15.02 ± 2.96	17.58 ± 3.70	20.65 ± 4.95	<0.001	17.75 ± 4.56
Cervical-SAT (cm^2^)	25.07 ± 7.18	32.83 ± 10.29	42.99 ± 12.63	<0.001	33.45 ± 12.54
Age (year)	46.80 ± 9.82	48.82 ± 9.11	47.67 ± 8.88	0.492	47.76 ± 9.28
Waist circumference (cm)					
Men	100.3 ± 5.3	106.4 ± 4.8	116.4 ± 7.3	<0.001	107.7 ± 8.9
Women	92.6 ± 6.4	96.2 ± 6.0	105.2 ± 12.5	0.004	98.3 ± 10.2
Diastolic Blood Pressure (mmHg)	78.42 ± 9.86	80.16 ± 9.79	83.45 ± 10.80	0.001	80.69 ± 10.34
Systolic Blood Pressure (mmHg)	122.13 ± 15.36	124.41 ± 14.71	129.11 ± 17.52	0.002	125.24 ± 16.13
Blood biomarkers					
Fasting Plasma Glucose (mg/dL)	105.71 ± 15.64	105.19 ± 16.31	110.68 ± 24.43	0.271	107.21 ± 19.34
Serum Insulin (μU/mL)	13.67 ± 7.25	16.13 ± 9.28	21.23 ± 12.19	<0.001	17.05 ± 10.27
HbA1c (%)	5.47 ± 0.48	5.45 ± 0.40	5.70 ± 0.61	0.006	5.54 ± 0.52
HOMA-IR	3.62 ± 2.16	4.21 ± 2.75	5.93 ± 3.92	<0.001	4.60 ± 3.18
Serum LDL-cholesterol (mg/dL)	120.04 ± 32.37	123.47 ± 30.39	121.82 ± 31.41	0.917	121.78 ± 31.32
Serum HDL-cholesterol (mg/dL)					
Men	42.12 ± 9.06	40.74 ± 9.26	41.98 ± 11.67	0.381	41.62 ± 10.04
Women	55.73 ± 16.23	61.18 ± 23.01	51.74 ± 6.95	0.933	56.05 ± 16.33
Serum triglycerides (mg/dL)	70.62 ± 41.58	73.44 ± 40.36	73.39 ± 42.35	0.723	72.48 ± 41.31
Abdominal fat depots					
Superficial-SAT (cm^2^)	106.1 ± 35.0	132.5 ± 47.0	186.0 ± 66.5	<0.001	141.7 ± 61.0
Deep-SAT (cm^2^)	161.2 ± 45.5	209.0 ± 48.3	278.3 ± 71.7	<0.001	216.4 ± 74.2
VAT (cm^2^)	141.5 ± 48.5	175.7 ± 60.5	209.1 ± 71.5	<0.001	175.6 ± 66.8

Tertiles were calculated based on equal division into three of study population by BMI values. Plus-minus values are means ± SD. *p* of trend was calculated using Kendall’s tau-b correlation test. Cervical adipose tissue indexes were log transformed to calculate *p* of trend. BMI, body mass index; HOMA, homeostasis model assessment; IR, insulin resistance; LDL, low-density lipoprotein; HDL, high-density lipoprotein; SAT, subcutaneous adipose tissue; VAT, visceral adipose tissue.

**Table 2:** 18 months changes in cervical and chin SATs across lifestyle intervention groups.

	LF Diet		MED/LC Diet		*p* Value LF: MED/LC ^1^	*p* Value LF ± PA: MED/ LC ± PA ^2^	Entire Change
	LF PA+	LF PA−	MED/LC PA+	MED/LC PA−			
6 months							
Chin-SAT	−1.0 cm^2^ ± 1.8 (−5.1% ± 9.7) ***		−1.1 cm^2^ ± 2.0 (−5.6 ± 11.5) ***		0.574 (0.603)	NA	−1.1 cm^2^ ± 1.9 (−5.3% ± 10.7) ***
Cervical-SAT	−5.3 cm^2^ ± 6.4 (−13.8% ± 15.1) ***		−5.1 cm^2^ ± 7.8 (−12.3% ± 18.4) ***		0.752 (0.603)	NA	−5.2 cm^2^ ± 7.2 (−13.1% ± 16.9) ***
18 months							
Chin-SAT	−0.1 cm^2^ ± 2.3	0.5 cm^2^ ± 2.1	−0.2 cm^2^ ± 2.3	−0.1 cm^2^ ± 2.4	0.428	0.390	0.0 cm^2^ ± 2.3 (0.8% ± 12.6)
	(0.2% ± 12.2)	(2.9% ± 12.1)	(−0.1% ± 12.2)	(0.5% ± 14.5)	(0.477)	(0.623)	
Cervical-SAT	−2.5 cm^2^ ± 7.3	−3.1 cm^2^ ± 8.5	−4.0 cm^2^ ± 6.6	−2.8 cm^2^ ± 7.1	0.398	0.482	−3.1 cm^2^ ± 7.3
	(−6.6% ± 20.0) **	(−7.0% ± 20.0) **	(−10.2% ± 17.0) ***	(−6.8% ± 18.2) **	(0.437)	(0.706)	(−7.8% ± 18.7) ***

Plus-minus values are means ± SD. 6-month: entire, *n* = 143; LF, *n* = 69; MED/LC, *n* = 74. 18-month: entire, *n* = 214; LF PA+, *n* = 59; LF PA−, *n* = 49; MED/LC PA+, *n* = 62; MED/LC PA−, *n* = 44. ^1^ *p* values for differences between the two diet groups were calculated by T-test or Mann-Whitney test. ^2^ *p* values for differences between the four intervention groups were calculated by analysis of variance or Kruskal-Wallis test Statistical significance: ** *p* ≤ 0.01, *** *p* ≤ 0.001; p values were calculated by paired T-test of the log transformed index. LF, low-fat; MED/LC, Mediterranean/low-carbohydrate; PA, physical activity. NA- not applicable at time 6, before PA randomization.

## Supplementary Materials

The following are available online at https://www.mdpi.com/article/10.3390/nu13113827/s1. File S1. Dietary and Physical Activity Intervention; File S2. Assessment of body fat depots; File S3. Laboratory Methods; File S4. Power calculation; Table S1. Baseline characteristics of the CENTRAL study population; Table S2. Associations between 18-month dynamics of cervical and chin SATs and dynamics of anthropometric measurements or body fat depots; multivariate regression models.
