# Supplementary material for: The Effect of Weight-Loss Interventions on Cervical and Chin Subcutaneous Fat Depots; the CENTRAL Randomized Controlled Trial
Source: Nutrients. 2021 Oct 27;13(11):3827. doi: 10.3390/nu13113827 (PMC8617936; doi:10.3390/nu13113827)
Supplement: Supplementary file 1 [file nutrients-13-03827-s001.zip › nutrients-1413944-supplementary.pdf]

Supplemental material File S1: Dietary and Physical Activity Intervention

The members of each diet group were divided into small group for nutritional counseling sessions. Clinical dietitians met with their groups every week during the first month of intervention, and then once a month for the remainder of the trial. Each session lasted 90-minutes and was conducted during the day in the workplace. To maintain equal intensity of treatment, the session's format and the quality of the materials were similar among the diet groups, except for instructions and materials specific to each diet strategy. We used telephone calls and text-messages to motivate participation. Both diets aimed at an energy intake of 1500 kcal/day for women and 1800 kcal/day for men, restricted trans-fat and refined-carbohydrates intake, while encouraging vegetables consumption. Lunch, which is typically the main meal in this population, was provided exclusively by the workplace cafeteria during the work week.

Low fat diet

The Low-fat, restricted-calorie diet was based on the revised American Heart Association guidelines [1,2]. We aimed for 30% of calories from fat, 10% of calories from saturated fat, an intake of 300 mg of cholesterol per day, and maximal dietary fibers consumption. The participants were counseled to consume low-fat grains, vegetables, fruits, and legumes, and to limit their consumption of additional fats, sweets, and high-fat snacks.

Mediterranean/low-carbohydrates diet

The MED/LC diet combined the Mediterranean and low-carbohydrate diets described in our previous weight loss trial (the DIRECT tria l[3]), but in the present study, the diets were calorie restricted; the diet restricted carbohydrate intake (to less than 40 g/day in the first two months, and thereafter a gradual increase up to 70 g/day) and increased protein and fat intake, mostly from vegetarian sources, according to the MED diet. The MED/LC diet was rich in vegetables and legumes and low in red meat, with poultry and fish replacing beef and lamb. This group was also provided with 28 g of walnuts/day [160 Kcal/84% fat, mostly PUFA (omega-3 α-linolenic acid)] starting from the third month.

Physical activity

Participants who were randomized to the PA intervention group (LF PA+ and MED/LC PA+) received a free 12-month gym membership, and monthly 60-minute PA workshop in the workplace, directed by a certified fitness instructor, who was blinded to the allocation of the diet. They were instructed to 80% aerobic exercise three times a week, gradually increasing from 20 minutes at 65% maximum heart-rate to 60 minutes at 80% maximum heart-rate[4,5]. The resistance training increased from one set of 60%

of the maximum weight up to two sets with 80% of the maximum weight [6]. Adherence was assessed by electronic monitoring of gym entries and self-reported questionnaire [7].

Electronic questionnaires

Adherence to the diet and PA was evaluated using a self-administered validated food-frequency questionnaire (FFQ), which included 127 food items and 3 portion size pictures for 17-selected food items, and physical activity questionnaire [7,8]. The electronic interface of the questionnaires ensured the completeness of data by prompting the participant when a question was not answered or not in the logical range. We also assessed symptoms, adverse-effects, quality of life, and medication usage.

Supplemental material File S2: Assessment of body fat depots

Abdominal fat depots

Abdominal fat depots, i.e. superficial-SAT, deep-SAT and VAT, were calculated using in-house MATLAB-based semi-automatic software [9] as the mean of three axial slices: L_2-L_3, L_4-L_5, and L_5-S_1. The superficialis fascia used to differentiate between deep-SAT and superficial-SAT. Quantification of the fat mass regions included the area of each fat type and its proportion (percentage) of the total area of all fat types. To obtain absolute measurements in metric units, a scaling procedure was applied before the segmentation to determine the real pixel dimensions. A comparison of 2D analysis with 3D analysis for 30 scans showed the 2D analysis to be highly accurate (r=0.97;p=0.001).

Intrahepatic fat

Intrahepatic fat percentage was quantified utilizing a region of interest technique, which is based on measurements of tissue densities [fat/fat + water], using the Fat Ratio Calculation PRIDE software package (Philips Healthcare). The lower cut-off point to initiate analysis was a hepatic area of 2000 mm^2. The number of ROIs in each slice (2D images at 3 cm intervals) was determined proportionally to the image area (1 ROI:2000 mm2). Each slice was divided into quarters, and ROIs were chosen in each of the four quarters such that the entire liver was represented. The mean percentage of fat for each slice and quarter were determined, and the mean percentage of fat in the liver was calculated.

Renal sinus fat

Renal sinus fat was analyzed by using in-house semi-automatic MATLAB-based program. The area (cm^2) of renal sinus fat was obtained from the middle slice of each kidney at the level of the 1st-2nd lumbar vertebra. In addition, renal sinus fat was measured by calculating the area of a polygon, which was defined by the dimensions of each kidney. Since the right kidney sits slightly lower than the left to accommodate the liver, a different slice was used for each kidney.

Femoral Intermuscular fat

Femoral Intermuscular fat was quantified a single 2D fat-phase axial slice from the mid-thigh of the right leg, from the femoral head to the medial and lateral condyle. Our semi-automatic MATLAB-based program was applied to distinguish between adipose and lean tissues and to calculate the area (cm^2) of femoral intermuscular adipose tissue. A 30% threshold was used automatically distinguish fat from non-fat, followed by manual delimitation of the different ROIs according to anatomical landmarks.

Pericardial fat

For the cardiac-MRI acquisition, scans were performed in a single-shot, breath-hold sequence using cardiac gating by the VCG technique (Vector Cardiac gating, Philips Medical Systems and merged all for volumetric values) in a two-block. Pericardial fat was analyzed from the level of the pulmonary trunk to the level of the apex of the heart in axial view. Reconstructing the imaging into slices gave an analysis range of 8 to 13 cm in a series of 16-26 slices on average. Using in-house semi-automatic MATLAB-based program, the first slice for each study was used for determination of the fat-recognition sensitivity threshold for the analysis of the specific series. This was done using the ROI method with a fixed-sized 2X2 pixels circle constantly placed on the fat tissue in the aortic recess (just anterior to the conjunction between the ascending aorta and the pulmonary trunk emerging from the ventricles). Upper and lower threshold values for fat tissue were set as 2.5 standard deviations from the average ROI-set value. Using the threshold-index, fat was automatically detected in each slice.

Inter and intra-observer correlations:

	Inter-observer correlation (2 independent observers)		Intra-observer correlations		
	r	p value	r	p value	n
Visceral fat	0.99	<0.001	0.99	<0.001	60
Hepatic fat	0.99	<0.001	0.98	<0.001	28
Renal sinus fat	0.97	<0.001	0.98	<0.001	28
Intermuscular fat	0.98	<0.001	0.99	<0.001	30
Pericardial fat (sub-study)	0.95	<0.05	0.99	<0.05	14 (280 slices)

Supplemental material File S3: Laboratory Methods

FPG was measured by Roche GLUC 3 (hexokinase method). Plasma insulin was measured with the use of an enzyme immunometric assay (Immulite automated analyzer, Diagnostic Products, coefficient of variation (CV)=2.5%). Homeostasis model assessments of insulin resistance (HOMA-IR) and of beta-cell function (HOMA-Beta) were calculated using the HOMA Calculator v2.2.3. Glycated hemoglobin (HbA1c) was measured with Tina-quant hemoglobin A1c Gen.3 (Roche). Serum total cholesterol (CV=1.3%), HDL-c, LDL-c, and triglycerides (CV=2.1%) were determined enzymatically with a Cobas 6000 Automatic Analyzer (Roche). Liver enzymes and bilirubin were measured with Roche Chemicals on the Cobas 6000 (Alkaline Phosphatase acc.). Plasma levels of high-molecular-weight adiponectin were measured by an enzyme-linked immunosorbent assay (ELISA) (AdipoGen or Axxora), with a coefficient of variation of 4.8%. Plasma leptin levels were assessed by ELISA (Mediagnost), with a coefficient of variation of 2.4%. Fetuin-A was measured by ELISAs (Biovendor, Heidelberg, Germany).

Supplemental material File S4: Power calculation

The primary outcome of this study was the effect of lifestyle intervention on dynamics of cervical subcutaneous fat as measured by MRI. As data referring to dynamics of cervical-SAT during lifestyle intervention are sparse, we estimated the expected change in cervical-SAT based on studies regarding changes in SAT[10] and interscapular fat[11] during lifestyle intervention. A previous study among 243 subjects at increased risk for type 2 diabetes assessed adipose tissue compartments through whole body MRI scans before and after 9 months of lifestyle intervention [10]. The observed changes in men were -15.1% in VAT, -11.3% in SAT, -10.9% in lower extremities fat and -12.4% in upper extremities fat. Another study among 172 subjects demonstrated -9.6% reduction in interscapular fat during lifestyle intervention. Based on these measurements we had expected an average decrease of 10% in cervical-SAT following 18 months of intervention. Power calculation was performed using WinPepi computer program version 11.39. Considering 2-sided significance level of 0.05, standard deviations of 15.72 before and 15.29 after the intervention, and a sample size of 200 participants, we had 90.6% statistical power to detect a 10% difference.

Supplemental material Table S1: Baseline characteristics of the CENTRAL study population

Characteristic	LF Diet (n=139)	MED/LC Diet (n=139)	All (n=278)
**Age (yr)**	48.36±9.23	47.41±9.32	47.88±9.27
**Male gender (n, %)**	122 (87.8%)	125 (89.9%)	247 (88.8%)
**Body Mass Index (kg/m^2)**	30.76±3.72	30.97±3.96	30.87±3.84
**Waist Circumference (cm)**	106.2±9.0	107.2±10.2	106.7±9.6
**Metabolic Syndrome Criteria (n)**	2.27±1.05	2.12±1.22	2.19±1.14
**Chin-SAT[3] (cm^2)**	17.99±4.57	17.50±4.56	17.75±4.56
**Neck-SAT[3] (cm^2)**	32.87±12.48	34.04±12.61	33.45±12.54
**Blood pressure**			
**Systolic Blood-Pressure (mmHg)**	125±15.1	125.2±17..1	125.1±16.1
**Diastolic Blood-Pressure (mmHg)**	79.4±9.8	81.7±10.9	80.5±10.4
**Blood biomarkers**			
**Fasting Plasma Glucose (mg/dL)**	106.52±17.54	108.09±20.91	107.31±19.28
**Serum Insulin (µU/ml)**	17.08±10.46	16.84±9.96	16.96±10.20
**HbA$_{1C}$ (%)**	5.53±0.54	5.56±0.49	5.54±0.51
**HOMA-IR**	4.54±3.00	4.62±3.33	4.58±3.16
**Serum LDL-cholesterol (mg/dL)**	123.82±31.93	120.83±30.86	122.33±31.38
**Serum HDL-cholesterol (mg/dL)**	43.24±12.98	43.04±10.20	43.14±11.65
**Serum triglycerides (mg/dL)**	75.05±42.78	70.15±39.45	72.59±41.14
**Abdominal fat depots**			
**Superficial-SAT (cm^2)**	142.6±62.2	142.4±64.0	142.5±63.0
**Deep-SAT (cm^2)**	212.8±68.8	220.0±79.5	216.4±74.3
**VAT (cm^2)**	178.9±68.8	171.8±64.1	175.4±66.4

1 Plus-minus values are means ± SD.
2 T-test and chi-square test were used to compare characteristics between the diet groups. There were no significant differences between the groups.
3 Cervical and chin adipose tissue indices were log transformed in order to calculate differences between intervention groups .

Supplementary material Table S2: Associations between 18-month dynamics of cervical and chin SATs and dynamics of anthropometric measurements or body fat depots; multivariate regression models

18-month changes of anthropometric measurements/body fat depots	β coefficient of 18-month changes	
	∆Chin-SAT	∆Cervical-SAT
**Anthropometric measurements**		
**∆Weight (kg)**		
Adjusted for age and sex	0.664***	0.697***
**∆Waist circumference (cm)**		
Adjusted for age and sex	0.509***	0.487***
Adjusted for age, sex and ∆VAT	0.307***	0.304***
**Body fat depots**		
**∆Abdominal Superficial-SAT (cm^2)**		
Adjusted for age, sex and ∆WC	0.347***	0.275***
Adjusted for age, sex and ∆VAT	0.282***	0.248***
Adjusted for age, sex and ∆Weight	0.129	0.015
**∆ Abdominal Deep-SAT (cm^2)**		
Adjusted for age, sex and ∆WC	0.331***	0.284***
Adjusted for age, sex and ∆VAT	0.224**	0.209**
**∆ Abdominal VAT (cm^2)**		
Adjusted for age, sex and ∆WC	0.415***	0.352***
**∆Hepatic fat (%)**		
Adjusted for age, sex and ∆WC	0.298***	0.371***
Adjusted for age, sex and ∆VAT	0.255***	0.357***
Adjusted for age, sex and ∆Weight	0.045	0.104
**∆Renal sinus fat (cm^2)**		
Adjusted for age, sex and ∆WC	0.197**	0.317***
Adjusted for age, sex and ∆VAT	0.147*	0.284***
Adjusted for age, sex and ∆Weight	0.033	0.138*
**∆Intermuscular fat (cm^2)**		
Adjusted for ∆WC	0.265***	0.205**
Adjusted for ∆VAT	0.253***	0.175**
Adjusted for ∆Weight	0.120*	0.002
**∆Extrapericardial fat (ml)**		
Adjusted for ∆WC	0.355**	0.243
Adjusted for ∆VAT	0.339**	0.275*
Adjusted for ∆Weight	0.198	0.076
**∆Intrapericardial fat (ml)**		
Adjusted for ∆WC	0.252	0.331*
Adjusted for ∆VAT	0.333**	0.387**
Adjusted for ∆Weight	0.084	0.176

[1] 18-month changes in cervical and chin SAT were calculated as the percent of change from the baseline. Statistical significance: *p≤0.05; **p≤0.01; ***p≤0.001.
[2] ∆Weight was adjusted only for age and sex due to collinearity. ∆Waist circumference was adjusted only for age, sex and ∆VAT due to collinearity. ∆Deep-SAT and ∆VAT were not adjusted for weight changes due to collinearity. Extrapericardial and Intrapericardial fat were not adjusted for age and sex due to small sample size (n=80).
[3] Hepatic fat was log transformed.
[4] WC, Waist circumference; SAT, subcutaneous adipose tissue; VAT, visceral adipose tissue.

REFERENCES

1. Krauss, R.M.; Eckel, R.H.; Howard, B.; Appel, L.J.; Daniels, S.R.; Deckelbaum, R.J.; Erdman, J.; Kris-Etherton, P.; Goldberg, I.J.; Kotchen, T.A.; et al. AHA Dietary Guidelines Revision 2000: A statement for healthcare professionals from the Nutrition Committee of the American Heart Association. *Circulation* **2000**, *102*, 2284–2299, doi:10.1161/01.CIR.102.18.2284.

2. Lichtenstein, A.H.; Lichtenstein, A.H.; Appel, L.J.; Brands, M.; Carnethon, M.; Daniels, S.; Franch, H.A.; Franklin, B.; Kris-Etherton, P.; Harris, W.S.; et al. Diet and Lifestyle Recommendations Revision 2006: A Scientific Statement From the American Heart Association Nutrition Committee. *Circulation* **2006**, *114*, 82–96, doi:10.1161/CIRCULATIONAHA.106.176158.

3. Shai, I.; Schwarzfuchs, D.; Henkin, Y.; Shahar, D.R.; Witkow, S.; Greenberg, R.; Golan, R.; Fraser, D.; Bolotin, A.; Vardi, H.; et al. Weight loss with a low-carbohydrate, mediterranean, or low-fat diet. *N. Engl. J. Med.* **2008**, *359*, 229–241, doi:10.1097/01.ogx.0000334740.32446.f3.

4. Ismail, I.; Keating, S.E.; Baker, M.K.; Johnson, N.A. A systematic review and meta-analysis of the effect of aerobic vs. resistance exercise training on visceral fat. *Obes. Rev.* **2012**, *13*, 68–91, doi:10.1111/j.1467-789X.2011.00931.x.

5. DONNELLY, J.E.; BLAIR, S.N.; JAKICIC, J.M.; MANORE, M.M.; RANKIN, J.W.; SMITH, B.K.; American College of Sports Medicine Appropriate Physical Activity Intervention Strategies for Weight Loss and Prevention of Weight Regain for Adults. *Med. Sci. Sport. Exerc.* **2009**, *41*, 459–471, doi:10.1249/MSS.0b013e3181949333.

6. HASKELL, W.L.; LEE, I.-M.; PATE, R.R.; POWELL, K.E.; BLAIR, S.N.; FRANKLIN, B.A.; MACERA, C.A.; HEATH, G.W.; THOMPSON, P.D.; BAUMAN, A. Physical Activity and Public Health. *Med. Sci. Sport. Exerc.* **2007**, *39*, 1423–1434, doi:10.1249/mss.0b013e3180616b27.

7. Shai, I.; Vardi, H.; Shahar, D.R.; Azrad, A.B.; Fraser, D. Adaptation of international nutrition databases and data-entry system tools to a specific population. *Public Health Nutr.* **2003**, *6*, 401–6, doi:10.1079/PHN2002445.

8. Shai, I.; Rosner, B.A.; Shahar, D.R.; Vardi, H.; Azrad, A.B.; Kanfi, A.; Schwarzfuchs, D.; Fraser, D.; study, D. Dietary evaluation and attenuation of relative risk: multiple comparisons between blood and urinary biomarkers, food frequency, and 24-hour recall questionnaires: the DEARR study. *J. Nutr.* **2005**, *135*, 573–579, doi:135/3/573 [pii].

9. Golan, R.; Shelef, I.; Rudich, A.; Gepner, Y.; Shemesh, E.; Chassidim, Y.; Harman-Boehm, I.;

Henkin, Y.; Schwarzfuchs, D.; Ben Avraham, S.; et al. Abdominal Superficial Subcutaneous Fat: A putative distinct protective fat subdepot in type 2 diabetes. *Diabetes Care* **2012**, *35*, 640–647, doi:10.2337/dc11-1583.

10. Machann, J.; Thamer, C.; Stefan, N.; Schwenzer, N.F.; Kantartzis, K.; Häring, H.-U.; Claussen, C.D.; Fritsche, A.; Schick, F. Follow-up Whole-Body Assessment of Adipose Tissue Compartments during a Lifestyle Intervention in a Large Cohort at Increased Risk for Type 2 Diabetes. *Radiology* **2010**, *257*, 353–363, doi:10.1148/radiol.10092284.

11. Thamer, C.; Machann, J.; Staiger, H.; Müssig, K.; Schwenzer, N.; Ludescher, B.; Machicao, F.; Claussen, C.; Fritsche, A.; Schick, F.; et al. Interscapular Fat Is Strongly Associated with Insulin Resistance. *J. Clin. Endocrinol. Metab.* **2010**, *95*, 4736–4742, doi:10.1210/jc.2009-1942.
